# Respiratory Epithelial Adenomatoid Hamartoma in a Young Female: A Diagnostic Dilemma

**DOI:** 10.7759/cureus.46453

**Published:** 2023-10-04

**Authors:** Abdul Mujeeb, Prathap Reddy Singavarapu, Lalitha Alekhya Chaganti, Abhishek Sai Seelamonthula, Rakesh Kotha

**Affiliations:** 1 Otorhinolaryngology, Government Medical College, Siddipet, Siddipet, IND; 2 General Practice, Government Medical College, Siddipet, Siddipet, IND; 3 Neonatology, Osmania Medical College, Hyderabad, IND

**Keywords:** ct scan of nose and paranasal sinuses, endoscopic excision, respiratory epithelial adenomatoid hamartoma, nasal polyps, hamartoma

## Abstract

Hamartomas are non-neoplastic tissue abnormalities commonly found in various organs but rarely in the upper aerodigestive tract. Respiratory epithelial adenomatoid hamartoma (REAH) is a rare benign proliferation affecting the nasal cavity and sinonasal tract. It often mimics other nasal masses, leading to diagnostic challenges. We present the case of a 25-year-old female with recurrent epistaxis and chronic bilateral nasal obstruction. Diagnostic endoscopy revealed a polypoidal mass, later confirmed as REAH through histopathological examination. CT scans demonstrated soft tissue opacity but no erosion of surrounding bony structures. The patient underwent endoscopic excision, and the excised mass exhibited characteristic histological features of REAH. Endoscopic excision with careful postoperative follow-up can lead to successful outcomes in REAH cases. A year of follow-up revealed no recurrence.

## Introduction

The term hamartoma was first used in 1904 to describe a tumor-like mass, but they are non-neoplastic malformations or inborn abnormalities of tissue development. Hamartomas are often seen in the lung, kidney, liver, and spleen but are uncommon in the upper aerodigestive tract [1]. Respiratory epithelial adenomatoid hamartoma (REAH) is a rare benign epithelial proliferation that predominantly affects the nasal cavity and sinonasal tract [2,3]. This rare benign lesion arising from Schneiderian respiratory epithelium (pseudo-stratified respiratory epithelium) was described as a specific clinicopathological entity by Wenig and Heffner in 1995 [4]. REAH, being a rare condition, is often misinterpreted as inverted papilloma (IP) or low-grade sinonasal adenocarcinoma, which requires aggressive surgeries. On the other hand, misinterpreting REAH as chronic sinus inflammation may lead to ineffective treatment. The diagnostic challenges faced with REAH are because of its similarity to other nasal masses such as nasal polyps, IPs, or low-grade sinonasal adenocarcinomas. This case study aims to provide a comprehensive analysis of a diagnosed case of REAH. In this report, we also discuss other nasal masses similar to REAH.

## Case presentation

A 25-year-old female patient presented with chief complaints of recurrent epistaxis and chronic progressive bilateral nasal obstruction for one year associated with bilateral nasal discharge. The patient had no history of nasal allergies, headaches, facial pain, swelling over the face, or previous nasal surgeries. Diagnostic nasal endoscopy revealed a pink polypoidal mass admixed with mucoid discharge in the floor of bilateral nasal cavities. Upon probing, it was found to bleed on touch and insensitive to pain, and it was determined to have arisen from the posterior part on the left side of the nasal septum, extending posteriorly into the nasopharynx and entering the opposite nasal cavity. Subsequently, a biopsy was done, and a small bit of tissue was taken from the mass and sent for histopathological study. The study reported it as a case of REAH. A non-contrast CT scan was performed on the paranasal sinuses, revealing soft tissue opacity in the bilateral middle and inferior meatus, extending posteriorly into the choana, and filling the nasopharynx. It showed a blocked bilateral osteomeatal complex associated with the left concha bullosa, mucosal thickening in the left maxillary sinus, and bilateral ethmoidal sinuses. There were no signs of erosion or destruction of the surrounding bony structures.

The patient was then scheduled for endoscopic excision. After obtaining informed consent, the mass was excised endoscopically under general anesthesia. During surgery, the mass was observed to be attached to the posterior part of the left side of the nasal septum with a pedicle. The mass was excised from its attachment, and electrocautery was used to stop the bleeding. The excised mass was then pushed into the nasopharynx and carefully delivered from the oral cavity. The procedure was uneventful, and the excised mass was presented for histopathological evaluation. The study reported histological features of respiratory ciliated epithelium with glandular stroma and multiple glands of varying sizes with no evidence of cellular atypia, and the lesion was identified as respiratory adenomatoid hamartoma. Based on the comprehensive clinical, radiological, and histopathological study, the patient was diagnosed as a case of REAH. Subsequently, the patient received appropriate treatment and was discharged. Follow-up after a year showed no recurrence.

## Discussion

REAH is a benign, glandular proliferation of the surface epithelium of the nasal cavity and paranasal sinuses [4]. REAH is usually seen in the age group of 30-90 years [5], which has a male preponderance, according to a case series where the ratio of male to female was 7:1 [6]. This is a rare presentation, as the patient in our case was 25 years old and female. They can arise anywhere in the sinonasal epithelium, but, usually, around 70% of REAH cases arise from the posterior nasal septum. However, they can also occur in other common sites such as the nasopharynx, lateral nasal wall, olfactory cleft, and frontal, maxillary, and ethmoid sinuses and are predominantly unilateral in presentation [7,8]. In our case, the patient had a bilateral nasal presentation, which is quite rare. Patients with REAH often present with non-specific clinical symptoms such as nasal obstruction, nasal stuffiness, hyposmia, epistaxis, rhinorrhea, headache, and post-nasal drip.

**Figure 1:**
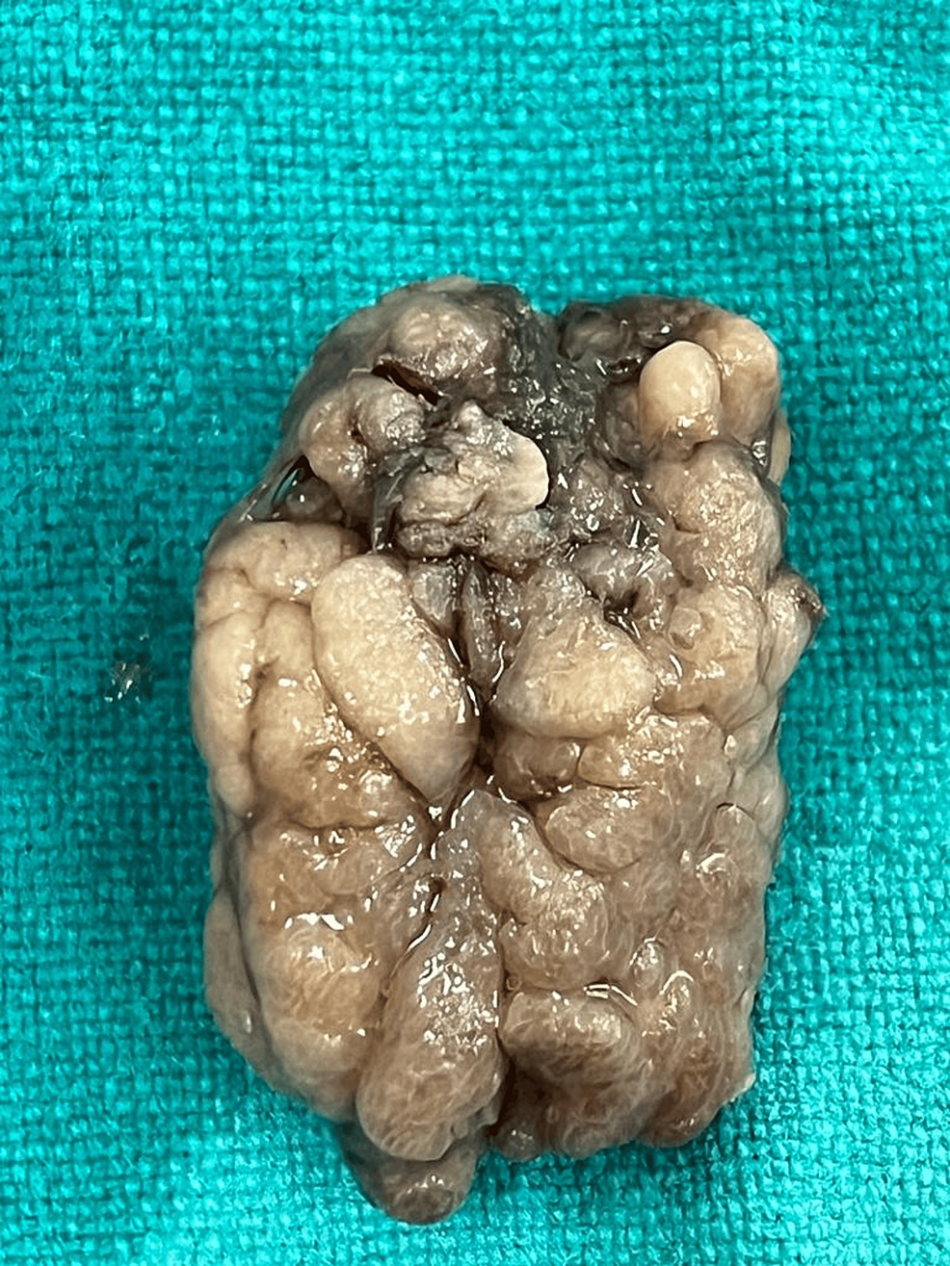
Gross polypoidal edematous and glistening appearance of respiratory epithelial adenomatoid hamartoma.

On gross examination, REAH appears to have a polypoidal appearance with a friable-to-firm consistency and a yellow-to-white color. Their appearance might also be edematous or glistening, as shown in Figure 1.

**Figure 2:**
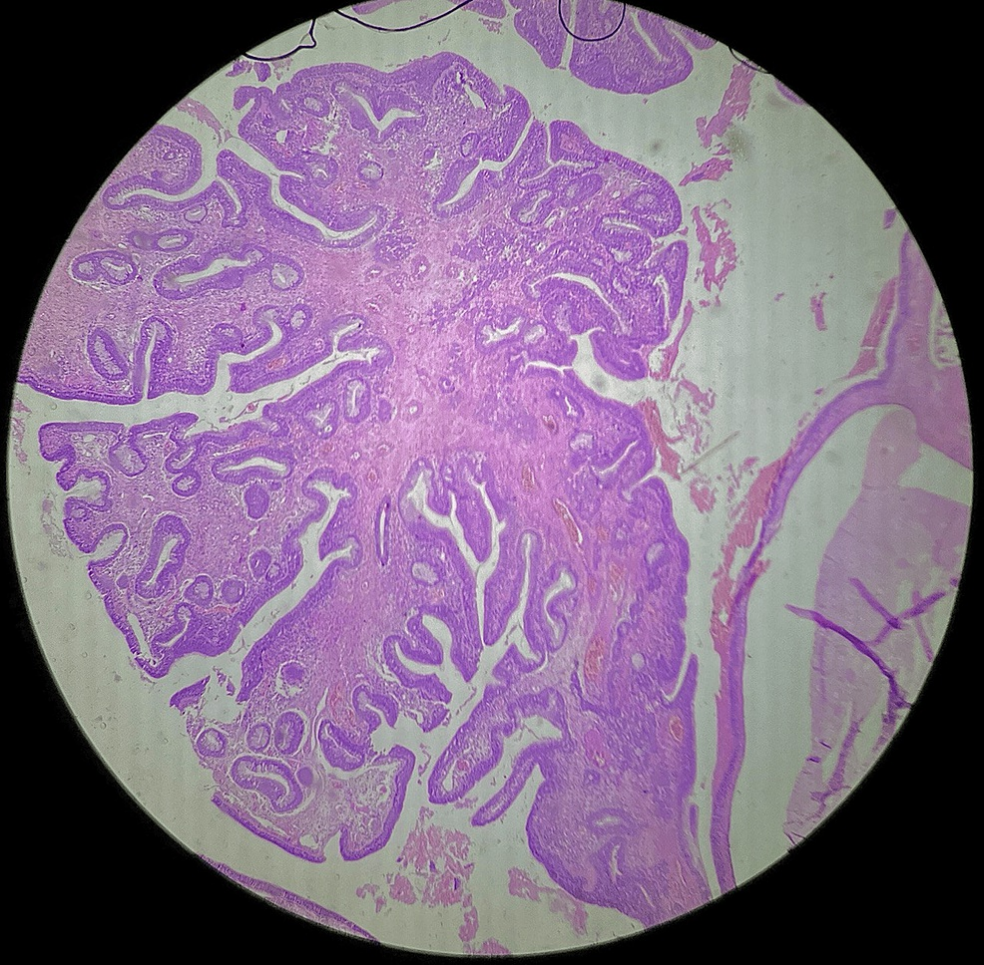
Microscopic picture of respiratory epithelial adenomatoid hamartoma under 4× magnification.

**Figure 3:**
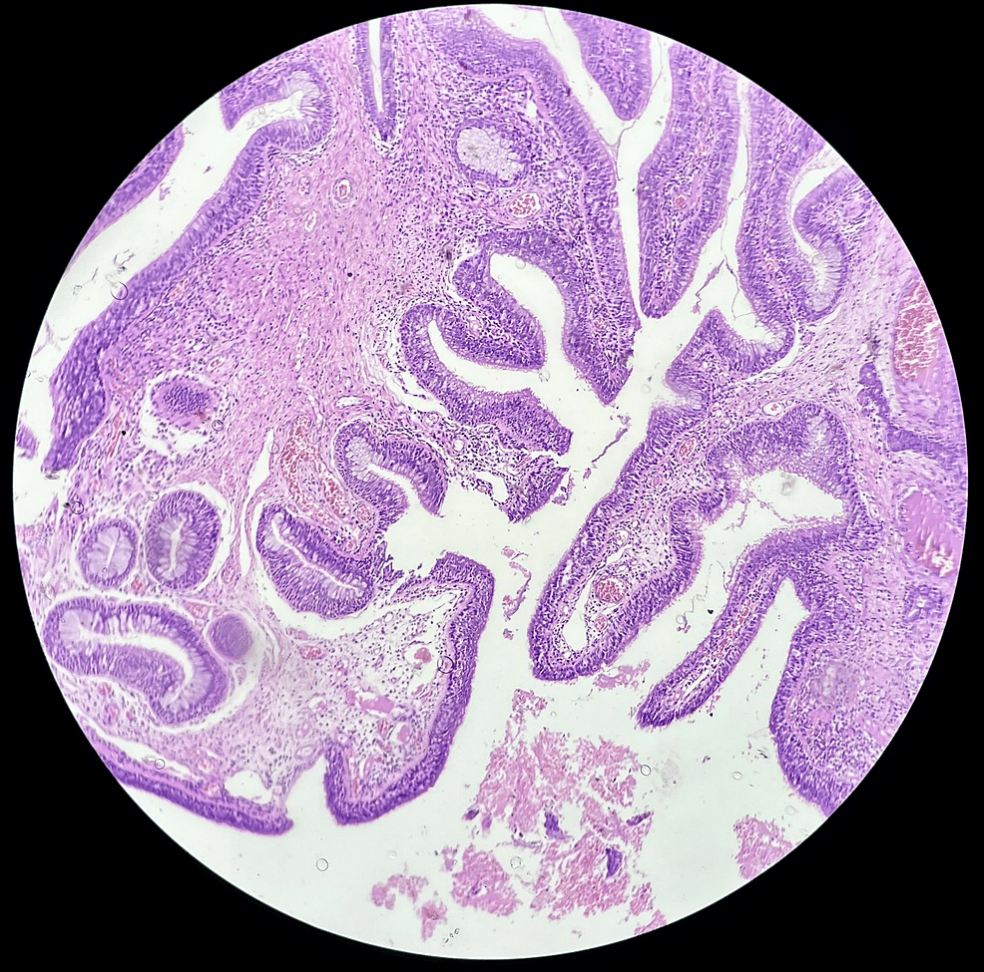
Microscopic picture of respiratory epithelial adenomatoid hamartoma under 10× magnification.

**Figure 4:**
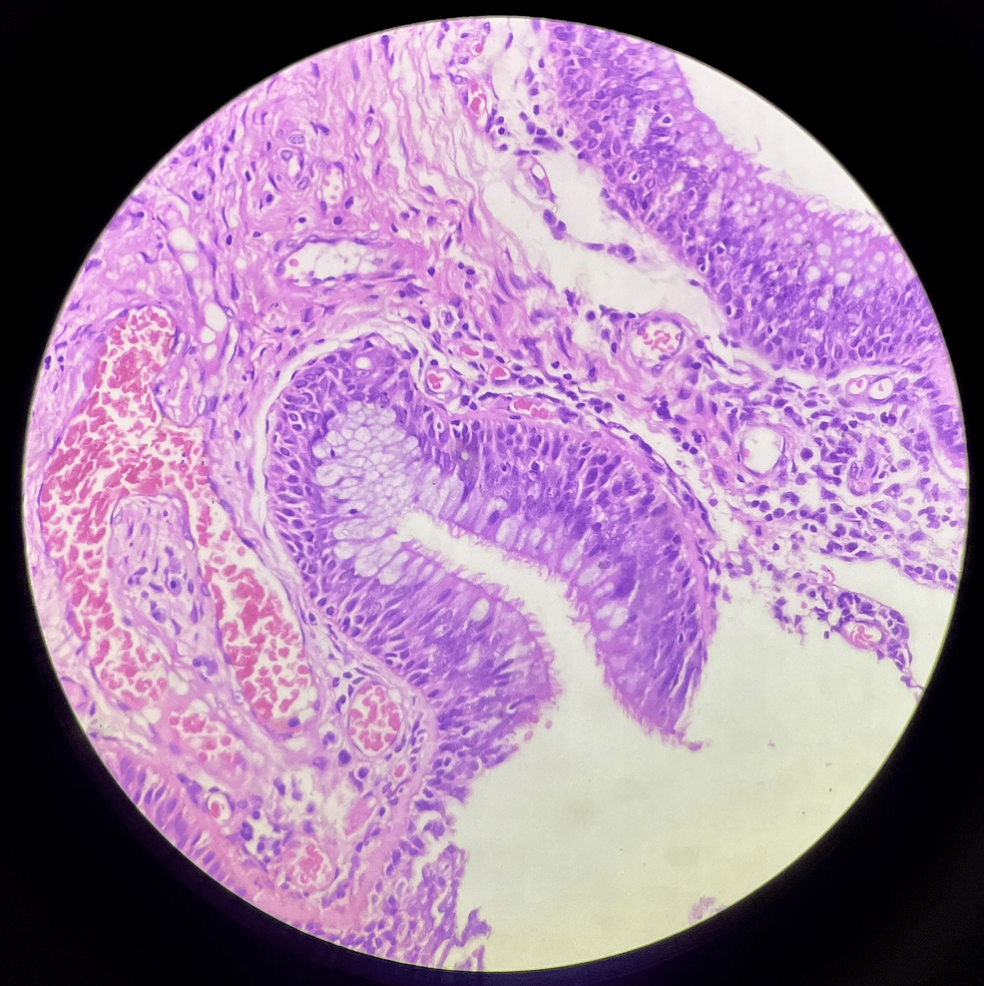
Microscopic picture of respiratory epithelial adenomatoid hamartoma under 40× magnification.

Histopathological reports of multiple serial and deeper sections from the nasal mass show surface epithelium lined by pseudo-stratified ciliated columnar epithelium with submucosal glandular proliferation of varying sizes, lined by ciliated respiratory epithelium (Figure 2). A few glands showing mucinous gland metaplasia and a lumen filled with eosinophilic material were also seen. Glands were lined by a thick eosinophilic basement membrane. There was no evidence of nuclear atypia. Stroma showing hyalinization, congested blood vessels, chronic inflammatory infiltrates, and benign serous mucous acini were noted (Figure 3). According to the study conducted by Rom et al., histopathological examination of nine samples showed glandular proliferation being prominent along with small to medium-sized, oval to round-shaped glands, which were separated widely by stromal tissue (Figure 4). The basement membrane was observed to be thickened along with edematous stroma [9]. The glands were also said to be cyst-like, dilated structures with mucinous, amorphous material within their lumen [4].

**Figure 5:**
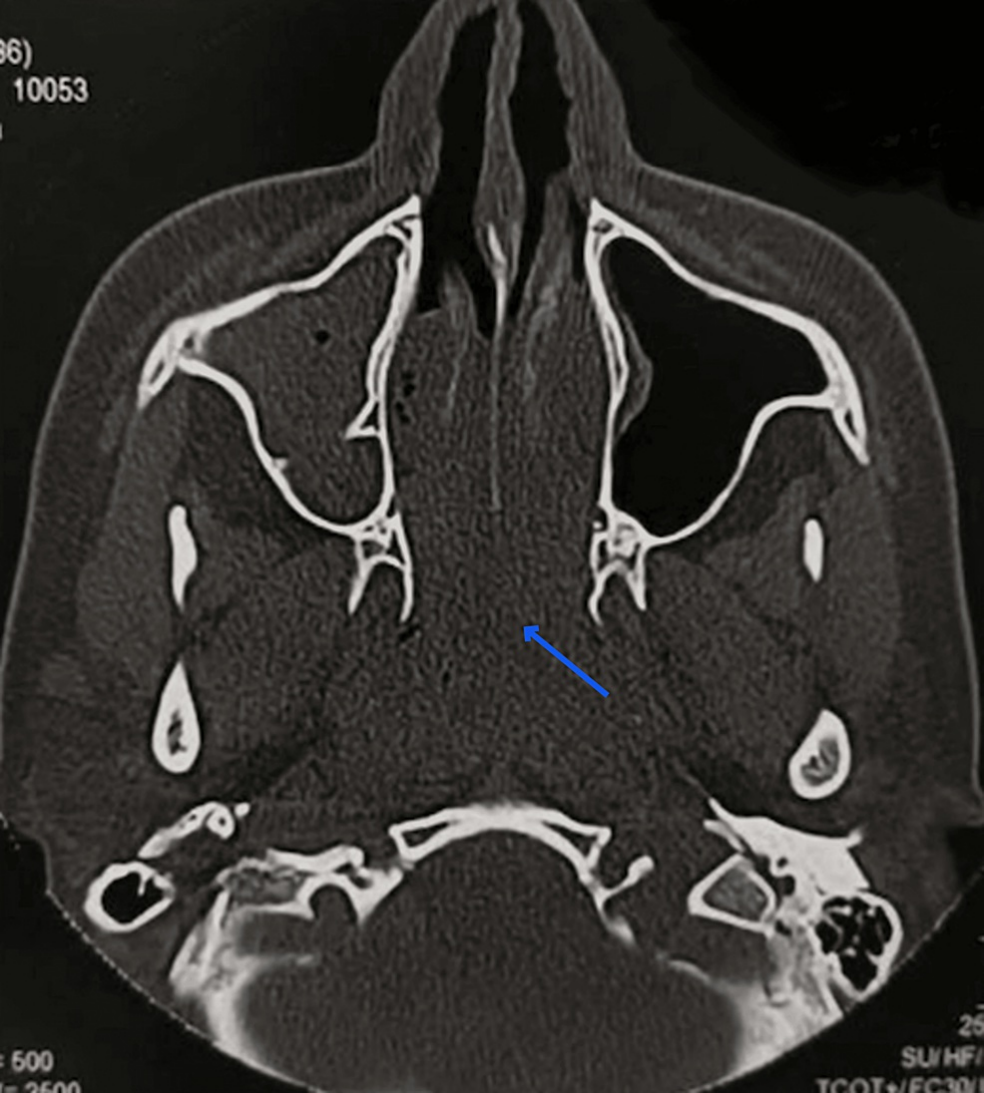
CT scan findings. The paranasal sinus shows right-sided maxillary sinusitis and soft tissue mass filling the bilateral inferior meatus and extending posteriorly into the nasopharynx. The blue arrow points toward the mass occupying the bilateral inferior meatus and extending into the nasopharynx.

**Figure 6:**
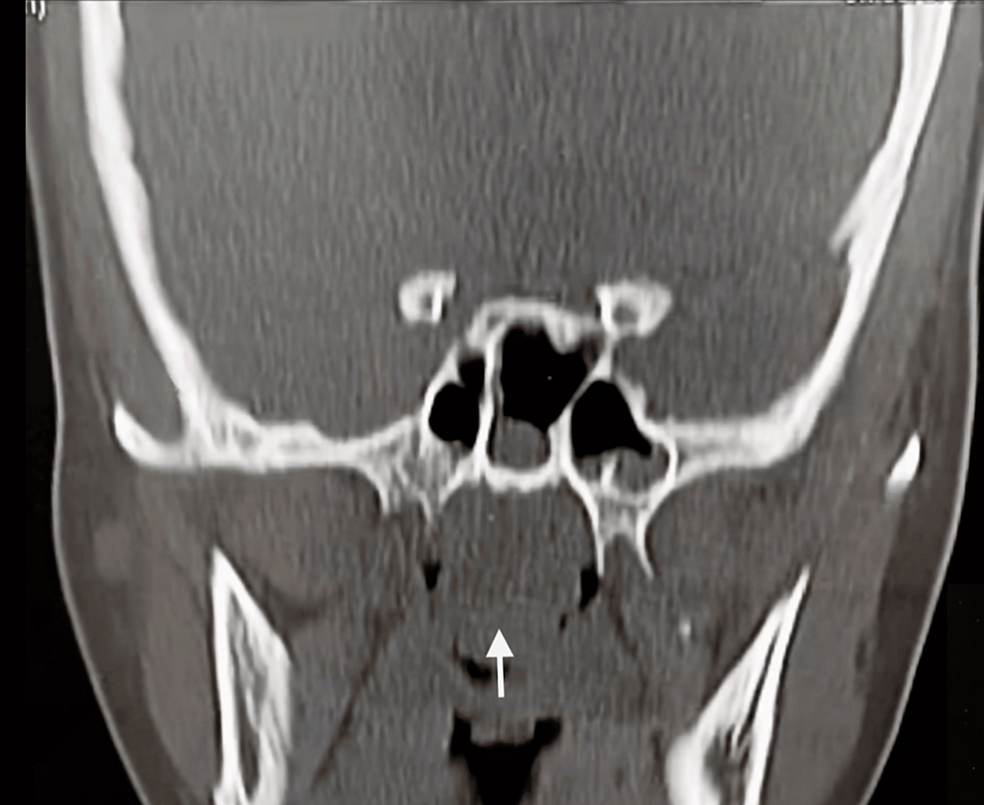
CT of the paranasal sinuses showing mucosal thickening in the right maxillary sinus and osteomeatal obstruction and soft tissue mass in the floor of bilateral nasal cavities. Arrow showing soft tissue opacity fills the choana bilaterally.

On radiological examination, common findings on a CT sinus of REAH are sinus opacification and their ostea being obstructed by a posterior nasal cavity with a polypoid lesion [10]. Another study found that the characteristic feature of REAH was the olfactory cleft being widened by greater than 10 mm on CT imaging [11]. In our study, CT scans of the paranasal sinus showed maxillary sinusitis, osteomeatal obstruction, and soft tissue mass in the floor of bilateral nasal cavities (Figures 5, 6).

Histopathological findings and CT imaging confirmed the diagnosis of REAH. Immunohistochemistry of REAH is usually positive for CK7, p63, and 34betaE12 and negative for CK20, CDX2, smooth muscle actin, calponin, and S100 protein [10]. As the diagnosis was confirmed directly by examination and histopathological findings, we did not perform immunohistochemistry in our study.

According to a recent study, REAH may be considered a benign neoplasm rather than a hamartoma [12]. For young adults presenting with nasal obstruction and epistaxis, it is important to consider a range of potential diagnoses, including inflammatory polyps, sinonasal adenocarcinomas, inverted papillomas, and adenoid hypertrophy.

Inflammatory nasal polyps are benign, inflammatory lesions that develop from the nasal and sinus mucosa. They are grape-like edematous masses that occlude nasal passages and result in symptoms such as nasal obstruction, nasal discharge, hyposmia, facial pain, and epistaxis [13]. Polyps can cause consequences such as sinusitis, asthma exacerbation, or obstructive sleep apnea in severe situations. The differential diagnosis of REAH and polyps is significant in some instances. Notably, the crucial distinction between REAH and inflammatory polyps is the involvement of the posterior nasal septal area in REAH.

Histologically, they exhibit many similar features, but the presence of more glandular proliferation of varied sizes, basement membrane thickening, and goblet cell metaplasia in our case favored the diagnosis of REAH. Histopathology of inflammatory nasal polyps reveals edematous stroma with inflammatory cell infiltrates, primarily eosinophils, lymphocytes, plasma cells, and mast cells, and histopathology also shows seromucinous gland proliferation [14]. Hence, it is significant to differentiate inflammatory polyps from REAH.

Low-grade sinonasal adenocarcinoma is another differential diagnosis for REAH. Low-grade sinonasal adenocarcinoma can originate from the surface epithelium and mucoserous glands of the submucosa [15].

To date, no guidelines for differentiating REAH (with a metaplastic mucinous gland component) from low-grade sinonasal adenocarcinoma have been made available. In the review of Wenig and Heffner’s description of this uncommon subtype, the authors discussed that a subset of lesions demonstrated florid mucinous gland metaplasia, with lumina filled with mucinous or amorphous material and stromal hyalinization seen surrounding glandular proliferation [4]. If a low-grade sinonasal adenocarcinoma originates from the surface epithelium or seromucous glands of the submucosa, distinction from a typical REAH is usually not problematic. The difficulty lies in the distinction of REAH with florid mucinous change from low-grade sinonasal adenocarcinoma arising from the surface epithelium, particularly in the setting of small biopsy samples. In addition to the lack of distinctive malignant features such as nuclear atypia, increased mitotic activity, and perineural invasion, the following features of REAH can be useful to differentiate: (1) the absence of complex back-to-back glandular architecture; (2) individual glands surrounded by eosinophilic basement membranes; (3) and the presence of ciliated epithelium lining the expanded lumens filled with mucin [16].

Capillary hemangioma closely resembles REAH, where both present with nasal obstruction, epistaxis, and bleeding with touch-on probing. Capillary hemangioma is distinguished from REAH based on the histopathological picture. A case study by Meshal et al. diagnosed a 30-year-old female based on radiological findings, which included contrast-enhanced CT findings that have shown heterogenous enhancement in multiple areas and MRI findings that have shown hypointense lesions on the non-contrast T1 image and heterogeneously hyperintense lesions with multiple flow voids in the T2 image. The mass showed inhomogeneous enhancement, particularly at the periphery, after contrast administration [17]. Histopathological picture of lobular capillary hemangioma shows vascular proliferation with inflammation and edema in the stroma, which is similar to the granulation tissue [18].

Olfactory neuroblastoma (ONB) arises from the olfactory epithelium and presents with unilateral nasal obstruction and epistaxis in most cases. Clinically, ONB is seen in the superior nasal cavity, which presents as a unilaterally arising large, polypoidal, glistening red-gray mass, often extending into the orbits, cranial vault, and paranasal sinuses [19]. ONB is diffusely positive for synaptophysin, chromogranin, NSE, and CD26, which provides an important diagnostic clue to differentiate from other diagnoses [20].

In our case, a simple endoscopic surgical excision was performed, followed by thorough postoperative care. Postoperative follow-up was done with nasal endoscopy, which showed no signs of recurrence.

## Conclusions

REAH, a rare benign hamartomatous growth in the sinonasal tract, is characterized by a respiratory epithelial lining and glandular stroma. It often manifests with clinical symptoms such as obstruction, epistaxis, and sinusitis. Fortunately, the treatment is straightforward: surgical excision, which typically yields excellent results. However, diagnosing REAH can be challenging for both surgeons and pathologists, as it requires careful differentiation from other neoplastic conditions such as adenocarcinoma and IP. Misdiagnosis can lead to extensive surgeries and postoperative procedures, underscoring the importance of raising awareness about this condition to prevent unnecessary patient morbidity.
